# Embryonic Lethality in Homozygous Human Her-2 Transgenic Mice Due to Disruption of the *Pds5b* Gene

**DOI:** 10.1371/journal.pone.0136817

**Published:** 2015-09-03

**Authors:** Carmen S. M. Yong, Janelle Sharkey, Belinda Duscio, Ben Venville, Wei-Zen Wei, Richard F. Jones, Clare Y. Slaney, Gisela Mir Arnau, Anthony T. Papenfuss, Jan Schröder, Phillip K. Darcy, Michael H. Kershaw

**Affiliations:** 1 Cancer Immunology Research Program, Sir Peter MacCallum Department of Oncology, University of Melbourne, Parkville, Victoria, 3010, Australia; 2 Department of Oncology, Wayne State University School of Medicine, Detroit, Michigan, United States of America; 3 Karmanos Cancer Institute, Detroit, Michigan, United States of America; 4 Molecular Genomics Core, Peter MacCallum Cancer Centre, East Melbourne, Victoria, 3002, Australia; 5 Bioinformatics and Cancer Genomics, Peter MacCallum Cancer Centre, East Melbourne, Victoria, 3002, Australia; 6 Bioinformatics Division, The Walter & Eliza Hall Institute of Medical Research, Parkville, Victoria, 3052, Australia; 7 Department of Medical Biology, University of Melbourne, Parkville, Victoria, 3010, Australia; 8 Department of Computing and Information Systems, University of Melbourne, Parkville, Victoria, 3010, Australia; 9 Department of Immunology, Monash University, Prahran Victoria, 3181, Australia; Baylor College of Medicine, UNITED STATES

## Abstract

The development of antigen-targeted therapeutics is dependent on the preferential expression of tumor-associated antigens (TAA) at targetable levels on the tumor. Tumor-associated antigens can be generated *de novo* or can arise from altered expression of normal basal proteins, such as the up-regulation of human epidermal growth factor receptor 2 (Her2/ErbB2). To properly assess the development of Her2 therapeutics in an immune tolerant model, we previously generated a transgenic mouse model in which expression of the human Her2 protein was present in both the brain and mammary tissue. This mouse model has facilitated the development of Her2 targeted therapies in a clinically relevant and suitable model. While heterozygous Her2^+/-^ mice appear to develop in a similar manner to wild type mice (Her2^-/-^), it has proven difficult to generate homozygous Her2^+/+^ mice, potentially due to embryonic lethality. In this study, we performed whole genome sequencing to determine if the integration site of the Her2 transgene was responsible for this lethality. Indeed, we report that the Her2 transgene had integrated into the *Pds5b* (precocious dissociation of sisters) gene on chromosome 5, as a 162 copy concatemer. Furthermore, our findings demonstrate that Her2^+/+^ mice, similar to Pds5b^-/-^ mice, are embryonic lethal and confirm the necessity for Pds5b in embryonic development. This study confirms the value of whole genome sequencing in determining the integration site of transgenes to gain insight into associated phenotypes.

## Introduction

Tumor cells often display an altered array of proteins that distinguish them from the cells of origin. On occasion, these proteins arise from oncogenic mutations, and are termed tumor-specific antigens (TSA), with expression on the tumor cells and conversely absent on the ‘normal’ surrounding tissue. These TSAs are generated *de novo* during oncogenic transformation of the cell and are not recognised as ‘self’ antigens by the immune system, and therefore have the potential to elicit an immune response. Furthermore, the restricted expression of these TSAs on tumor cells allow for the generation of specific therapeutic agents with little on-target/off-tumor effects. However, many antigens are also expressed on normal tissues to varying degrees, and are termed tumor-associated antigens (TAA). Cellular dysregulation that occurs during the oncogenic process can also lead to upregulation of normal tissue proteins. Whilst still present on the tissue cells of origin, this increased level of protein expression can sometimes be correlated with patient prognosis, and therefore can be identified as a TAA [1]. Such is the case in breast cancer, where the human epidermal growth factor receptor 2 (Her2/ErbB2) protein found on normal breast tissue is upregulated in 15 to 30% of breast cancers [2,3]. Moreover, overexpression of Her2 has been reported on many other types of cancer, including gastric cancer, non-small cell lung cancer and head and neck squamous cell carcinoma [4–6].

As Her2 is not exclusively present on breast tissue but is also found in the brain, the lower intestine and lung, immunotherapies directed against the Her2 antigen may also encounter on-target/off-tumor effects resulting in toxicity and autoimmunity [7]. In addition, the use of immunotherapies specifically targeting tumors arising from self-antigens has to overcome the issue of tolerance. As the most potent and reactive immune cells recognising self-antigens are deleted during positive selection, the pool of potentially reactive immune cells that are able to survive this process possess ‘sub-optimal’ avidity to their antigen compared to immune cells specific for antigens generated *de novo* [8].

Multiple mouse models of the TAA Her2 have emerged and facilitated the study and development of Her2 therapeutics. Two of the most commonly used promoters used to drive the expression of the Her2 antigen are the mouse mammary tumor virus (MMTV) and the whey acidic protein (WAP) promoter. Whilst both are highly expressed in the mammary gland, expression of the MMTV promoter has also been found in other organs, including the kidneys, lungs, testes as well as in T cells [9,10]. Similarly, low levels of the WAP promoter have been observed in the cerebrum, liver and kidney [11]. Unlike other ubiquitously expressing promoters often used in transgenic models, the expression of both the MMTV and WAP promoters are hormonally regulated, with altered expression occurring during hormonally regulated events such as pregnancy or mammary gland development [12]. In particular, the MMTV promoter has been used in multiple models, driving the expression of both the human Her2 antigen (MMTV-Her2) as well as the more commonly used Her2/neu antigen (MMTV-Her2/neu) [13–15]. The MMTV-Her2/neu model has been used extensively and has been integral in the development of breast cancer therapeutics. However, the differences between the homologues of the rat (neu) and human Her2 have made assessment of autoimmune and on-target/off-tumor effects hard to determine [16,17].

In order to adequately assess potential therapies in which the Her2 antigen could be targeted specifically on tumor cells, leaving the surrounding normal tissues with basal expression unaffected, it was necessary to generate a clinically relevant mouse model. Piechocki *et al*. therefore generated the human Her2 (Her2) transgenic mouse model driven by the whey acidic protein (WAP) promoter [18]. In this model, basal expression of the human Her2 antigen was constitutively expressed in the cerebellum and breast tissue. In addition, the expression pattern of the WAP promoter has been well characterised and utilized in driving transgene expression in multiple mouse models [11,19–21]. The generation of the WAP-Her2 mouse model (herein referred to the Her2 model) facilitated the development and testing of immunotherapies targeting the Her2 antigen on tumors in the presence of basal Her2 expression on some normal tissue. As the development of autoimmunity and on-target/off-tumor effects in patients is a major side effect in immunotherapy, the Her2 model has facilitated the development of safer and more clinically relevant therapies in a physiologically relevant immune-tolerant model. The on-target/off-target toxicity and autoimmunity related side effects of Her2 targeted therapeutics could now be adequately assessed.

Primary analysis of the Her2 mouse model has demonstrated its tolerance to Her2^+^ tumors. Conversely, Her2 tumors are often rejected in wildtype mice due to the high level of immunogenicity against the Her2 antigen. In addition, DNA vaccination with the Her2 antigen prior to Her2^+^ tumor challenge induced a robust anti-tumor response, with up to 33% survival of Her2^+^ mice (compared to 0% survival in Her2^+^ unvaccinated mice), indicating these mice were able to overcome tolerance to the Her2 antigen, and successfully mount an immune response. No autoimmunity or off-target responses were recorded in this study [18].

The tolerance to Her2 tumors in this mouse model has been critical in the development of immunotherapies against the Her2 antigen, particularly in solid established tumor models. The adoptive transfer of T cells genetically modified with a chimeric antigen receptor specific for the Her2 antigen into Her2 tumor bearing mice have demonstrated both tumor regression and prolonged survival in the absence of autoimmunity [22,23].

The generation of transgenic mice often involves the random integration of the desired transgene into the genome. While this integration often has little effect on the normal phenotype of the mice, there may be occasions where transgene integration could potentially disrupt a gene integral for development and survival. Heterozygous Her2 mice (Her2^+/-^) mice display a normal phenotype and are similar to their wild type littermates. However, transgenic mice are often bred in a homozygous state, ensuring the progeny themselves are transgenic and thereby reducing the variability between breeding pairs of heterozygous matings. Attempts at generating homozygous Her2^+/+^ mice have been unsuccessful, potentially since the inheritance of the two Her2 transgenes in this model resulted in embryonic lethality. As no overt pathology or abnormalities were observed in heterozygous mice, we speculated that it would be unlikely that the inheritance of twice as many Her2 copies would result in this lethality. Rather, we hypothesised that perhaps the integration of the Her2 transgene had interrupted a gene essential for embryonic or fetal development. To answer this, we performed whole genome sequencing (WGS) on Her2^+/-^ mice to determine the integration site of the transgene. Whole genome sequencing revealed the integration of the Her2 transgene had indeed interrupted a gene, Pds5b, whose function is known to be integral in the segregation of chromosomes in both meiosis and mitosis [24]. Surprisingly, we report that Her2^+/+^ mice display greater development defects than previously reported for some Pds5b^-/-^ mice [25,26]. Our findings further characterise the Her2 transgenic mouse model and validate WGS as an efficient method for determining transgene insertion. In addition, our findings support previous reports for the role of Pds5b in embryonic development.

The genetic characterization of Her2 mice described here forms a chapter in a PhD thesis with publications by Carmen S.M. Yong. Additional chapters will describe the use of these mice in the investigation of therapies for Her2^+^ cancers.

## Methods

### Mouse model

Ethics statement: This study was carried out in strict accordance with the recommendations of the Victorian Bureau of Animal Welfare, Department of Primary Industries, and the National Health and Medical Research Council's Australian code of practice for the care and use of animals for scientific purposes. The protocol was approved by the Institutional Animal Care and Use Committee: Peter MacCallum Cancer Centre Animal Experimentation Ethics Committee under Permit number E498. All efforts were made to minimize suffering. Mice were monitored daily for deterioration in condition and signs of stress, as defined by lethargy, ruffled fur or a hunched appearance, at which time the mice were considered to have reached the ethically permitted humane endpoint criteria and were humanely euthanized. Mice were euthanized using carbon dioxide asphyxiation. Tumors in excess of 150 mm^2^ were also considered to have reached humane endpoint criteria. Tumors with unhealed weeping ulcers involving fluid loss were also considered to have reached humane endpoint criteria, but mice with healed scabbed ulcers did not reach humane endpoint criteria and were permitted to survive.

The human Her2 transgenic mouse was previously generated by Piechocki *et al*. [18]. Mice were bred and maintained under specific pathogen-free conditions within the animal experimentation facility at the Peter MacCallum Cancer Centre. The mouse strain, given the name B6.Cg-Tg(Wap-ERBB2)229Wzw/J at Jackson Laboratories (stock number 010562), is referred to herein abbreviated form of Her2 mice.

### Cell culture and tumor studies

The murine 24JK fibrosarcoma cell line was kindly donated by Dr. Patrick Hwu (NIH, Bethesda, MD) [27] and maintained at 37°C in 5% CO_2_ in RPMI-1640 media supplemented with 5% heat-inactivated fetal calf serum (FCS) with 2 mM glutamine, 1 mM sodium pyruvate, 0.1 mM nonessential amino acids, 100 U/mL penicillin and 100 ug/mL streptomycin (Life technologies). The murine E0771 (LMC variant) breast adenocarcinoma line was kindly donated by Prof. Robin Anderson (Peter MacCallum Cancer Centre, Victoria, Australia) and maintained at 37°C in 10% CO_2_ in Dulbecco's modified Eagle medium (DMEM), supplemented as above. Both cell lines were retrovirally transduced with the human Her-2 antigen (ERB) as previously described [28].

1 x 10^6^ 24JKERB or 1 x 10^5^ E0771ERB were injected into Her2^+/-^ mice subcutaneously or 5 x 10^5^ E0771ERB injected into the mammary fat pad. Tumors were measured on days as stated and mice were sacrificed when tumor size reached ethical limit of 150 mm^2^.

### Generating Her2^+/+^ mice

Heterozygous Her2^+^ mice were interbred to generate litters consisting of Her2^-/-^, Her2^+/-^ and Her2^+/+^ mice. Embryos were harvested from pregnant Her2^+/-^ females from E14 onwards and analysed for signs of malformation and abnormal development. Tail clippings were taken at this time point and analysed for the presence of the Her2 transgene via polymerase chain reaction (PCR).

### Whole genome sequencing

Genomic DNA was extracted from the tails of Her2^+/-^ transgenic mice using the DNeasy Blood and Tissue Kit (Qiagen) according to manufacturers’ instructions or using phenol:chloroform extraction. The DNA was quantified and purity verified. 500 ng of DNA were fragmented using a focal acoustic device (Covaris S2) and used to prepare libraries with the KAPA Library Preparation Kit for Illumina platforms (KAPABIOSYSTEMS). Libraries were size selected to an average fragment size of 600 bp using the PippinPrep Instrument (SAGE Science). Three indexed libraries were pooled and sequenced across three lanes of an Illumina HiSeq2500 flowcell using High Output chemistry v3 (Illumina).

The sequencing data consisted of 100 nucleotide long paired-end reads with over 170 million fragments (2*172,481,778 reads). As a target for sequence alignment we concatenate the mm10 build of the mouse reference (http://genome.ucsc.edu) and the putative Her2 transgene sequence (as outlined by Wei *et al* [18]). The sequence mapping was performed by Bowtie2 (version 2.2.3 [29]). Bowtie2 is run with the “--local” option to enable it to partially map reads, which is needed for subsequent structural variant analysis. The alignment successfully placed 343,750,783 of the reads, corresponding to a mapping rate of 99.6% and average haploid genome coverage of 11.5.

### PCR genotyping

Genomic DNA of Her2^+/+^, Her2^+/-^ and Her2^-/-^ was isolated from tail clippings of E14.5 to E19 pups as previously mentioned. Briefly, genomic DNA was extracted from tissue samples (~1–2 mm^2^) using QuickExtract DNA extraction solution (Epicentre Technologies, Madison, WI, USA). After adding 20 μL QuickExtract solution to each tissue sample, the samples were incubated at 65°C for 20 minutes, then 95°C for a further 20 minutes. The samples were then diluted 1:10 in sterile deionised water, and 1 μL was used for PCR. PCR amplification was performed using the following primers; Pds5b Forward 5’ GGACTATTTACAGGAAACGTC 3’, Pds5b Reverse 5’ AGCAAGCCACCAGTAAACG 3’ and Her2 Forward 5’ GTCACAGGGGCCTCATCC 3’. The Her2 forward primer spanned the junction point of insertion of the transgene concatemer into the chromosome, with the first 14 bp annealing to the truncated 3’ end of the transgene, and the final 4 bp annealing to the Pds5b gene at insertion. DNA was amplified with the following conditions; 95°C for 5 minutes, 95°C for 30 seconds, 58°C for 30 seconds, 72°C for 30 seconds, repeat step 2 to 4 for 35 cycles, 72°C for 5 minutes. Amplified DNA was analysed on a 1% agarose gel.

## Results

### A range of Her2^+^ tumors grow progressively in Her2 mice

**Fig 1:**
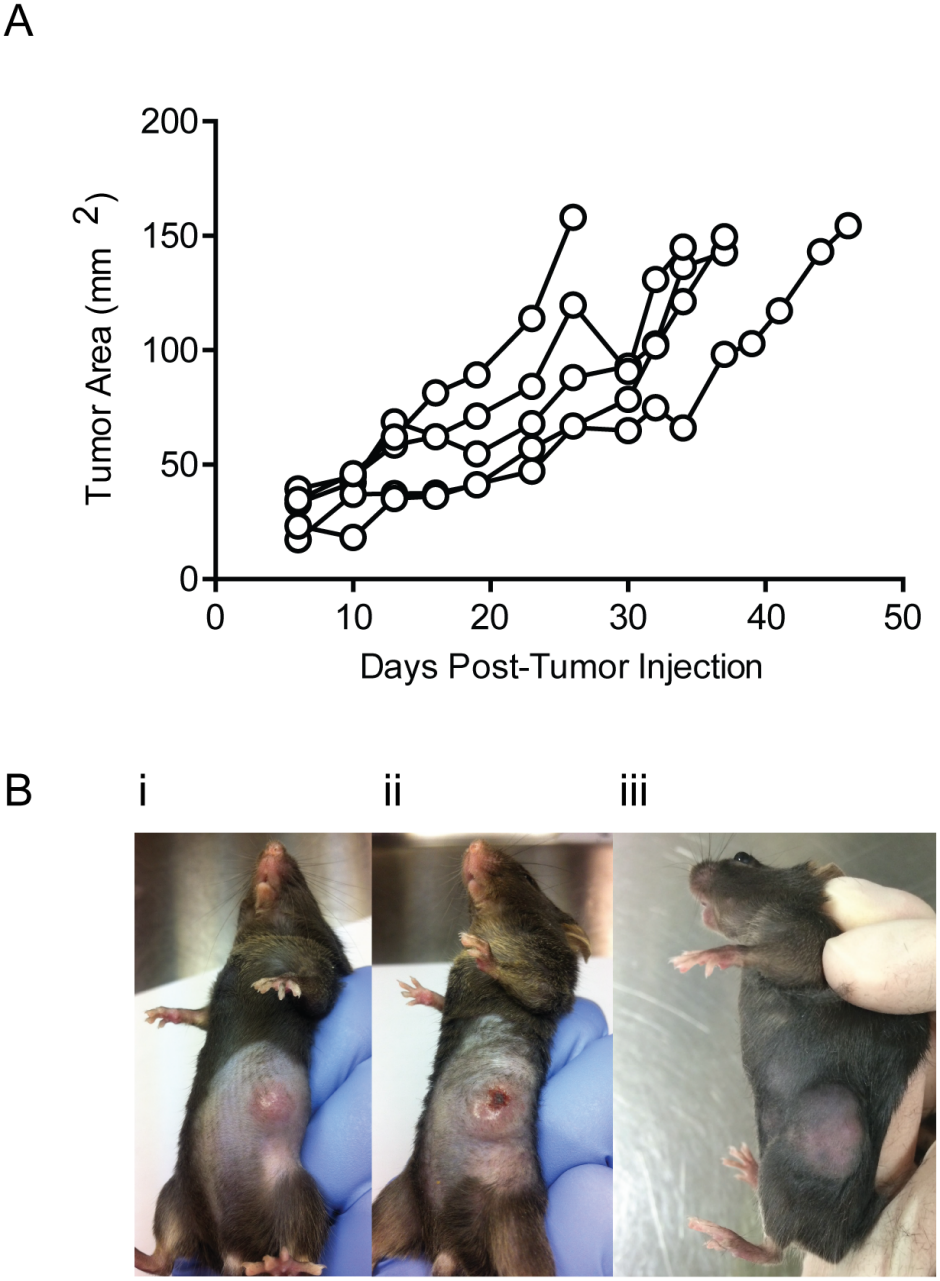
Human Her2^+^ tumors grow progressively in Her2 transgenic mice. Tumor measurements and images of Her2^+^ tumors growing in Her2^+^ mice. **A**) 1 x 10^6^ 24JKERB cells were injected subcutaneously (SC) into the flank of Her2^+^ mice. Growth curves of individual mice are represented. Mice were sacrificed when tumor volume reached the ethical limit of 150 mm^2^. **B)** Representative images of tumors following injection of 1 x 10^5^ E0771ERB cells were injected SC into the **i,ii)** flank or **iii)** 5 x 10^5^ cells into the mammary fat pad of Her2^+^ mice.

Human Her2^+^ tumors have been shown to grow poorly in wild type immunocompetent mice due to the highly immunogenic nature of the antigen. The expression of the human Her2 antigen from birth in the Her2 transgenic mouse models generated a functioning immune system tolerant to the Her2 antigen. As a result, Her2^+^ tumors were able to sufficiently evade the adaptive immune system and grow without hindrance. To validate tolerance of Her2^+/-^ mice, we first demonstrated the ability of Her2^+^ tumors to grow in Her2 transgenic mice. The murine sarcoma cell line 24JKERB consistently grew subcutaneously in Her2 transgenic mice (Fig 1A). Progressive growth of Her-expressing tumor cells was also confirmed using a murine breast cancer cell line, E0771ERB expressing human Her2. Subcutaneous E0771ERB injection leads to large tumors (Fig 1B(i)) that sometimes formed a scabbed ulceration (Fig 1B(ii)). Injection of E0771ERB cells into the 4^th^ mammary fat pad consistently led to the development of large mammary tumors (Fig 1B(iii)).

### Her2 mice produce a lower than expected frequency of transgenic progeny

Prior to this investigation, the standard method for detection of positive Her2 transgenic mice was to analyze for the presence of the Her2 transgene. As such, there was no precise method for detecting heterozygous or homozygous mice, as only transgenic positive mice could be identified. Genotyping of Her2 transgenic mice revealed an altered Mendelian ratio to what would normally be expected from a heterozygous mating. We noted over multiple generations the inheritance of the Her2 transgene was only present in approximately 50% of the progeny (239 out of 500 progeny or 48%), instead of the expected 75%, suggesting perhaps a complete absence of homozygous mice. To further substantiate our hypothesis, we had previously attempted generating homozygous mice through intensive iterative breeding programs however were never able to achieve 100% transgenic progeny. As no overt pathology in terms of development or immune function was noted in heterozygous mice, we hypothesised that perhaps the transgene integration site had interrupted an integral gene. We decided to utilize whole genome sequencing (WGS) to determine the integration site, as it is a fast, efficient and reliable method.

### Whole genome sequence analysis of the Her2 transgenic mice

**Fig 2:**
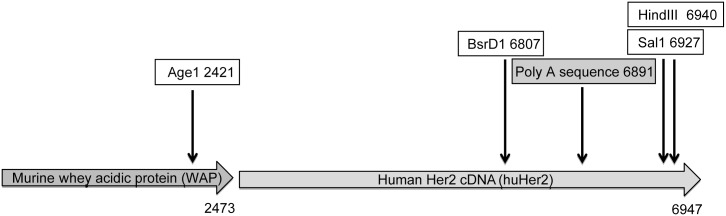
Transgene schematic and sequence. Schematic of the human Her2 cDNA transgene under the control of the whey acidic protein (WAP) promoter. The WAP-Her2 transgene was generated as previously described [18].

The generation of transgenic mouse models often involves the microinjection of the linearised DNA transgene into oocytes at the pro-nuclear stage. For most transgenes, this integration occurs in a non-homologous manner, as often these transgenes are preceded by a highly active promoter thus the transcriptional activity of the surrounding genome (with the exception of silencing epigenetic regulation) is irrelevant to the transgene expression. The Her2 mice were previously generated in such a manner, where the 2.5 kb whey acidic protein (WAP) promoter driving the 4.4kb c-ErbB2 transgene cDNA was digested to create a linearized transgene fragment, which was then microinjected into C57BL/6 oocytes ([18]) (Fig 2). The 6.9 kb sequence published from this study was used as a reference sequence to determine the integration point of the transgene into the genome using WGS. Our final sequence of the WAP-Her2 transgene was 6884 nt in length. The full sequence is listed in S1 Fig.

### Comparison of the WAP-Her2 sequence

We observed some non-Her2 sequences using WGS. A 34 nucleotide insert (CTGGGGATCCTCTAGAGTCGACCTGCAGGCA-TGC) at the 5’ end of the WAP promoter was found using WGS analysis (S1 Fig). A nucleotide BLAST search revealed this sequence to be remnants of a cloning vector used to generate the WAP-Her2 transgene. A 36 nucleotide section (AGGGGAGGTAACCCTGGCCCCTTTGGTCGG-GGCCCC) between the WAP promoter and Her2 5’ untranslated region (UTR) during our WGS analysis was determined to be homologous to the 5’ UTR of a novel variant of human Her2 cDNA as reported by Yamamoto *et al*. ([16]) (Genbank X03363) but absent from variant 1 of the huHer2 cDNA (Refseq NM_004448). Interestingly, a 61 nucleotide section (ATGA**AATAAA**GACCCAGGGGGAGAAGCTGGGATCCTCTAGAGTCGACGCATGCAAGCTTNA) at the 3’ region of the Her2 3’ UTR was absent in our WGS analysis. This 61 nucleotide sequence at the 3’ region of the Her2 3’ UTR contained a poly-adenylation sequence, **AATAAA** (bolded above). As such, the WAP-Her2 transgene found by WGS is lacking an endogenous 3’ poly-adenylation sequence, and it is likely the gene uses a poly-A sequence downstream of the integration site.

### The WAP-Her2 transgene is upstream of exon 3 in the Pds5b gene on chromosome 5

To derive the exact transgene sequence we first looked for aberrations in the mappings around the transgene, and refined the sequence to correct for such deviance. This iterative process of creating new transgene models and remapping to a new reference including this model yielded a final sequence, which was used in the following steps. The next step was to detect the insertion point(s) of the transgenic material into the native DNA of the mice. We performed structural variation analysis on the alignments using Socrates [30] and used Control-FREEC (version 6.7 [31]) for genome wide copy number analysis to establish the number of transgene instances inserted into the DNA.

**Fig 3:**
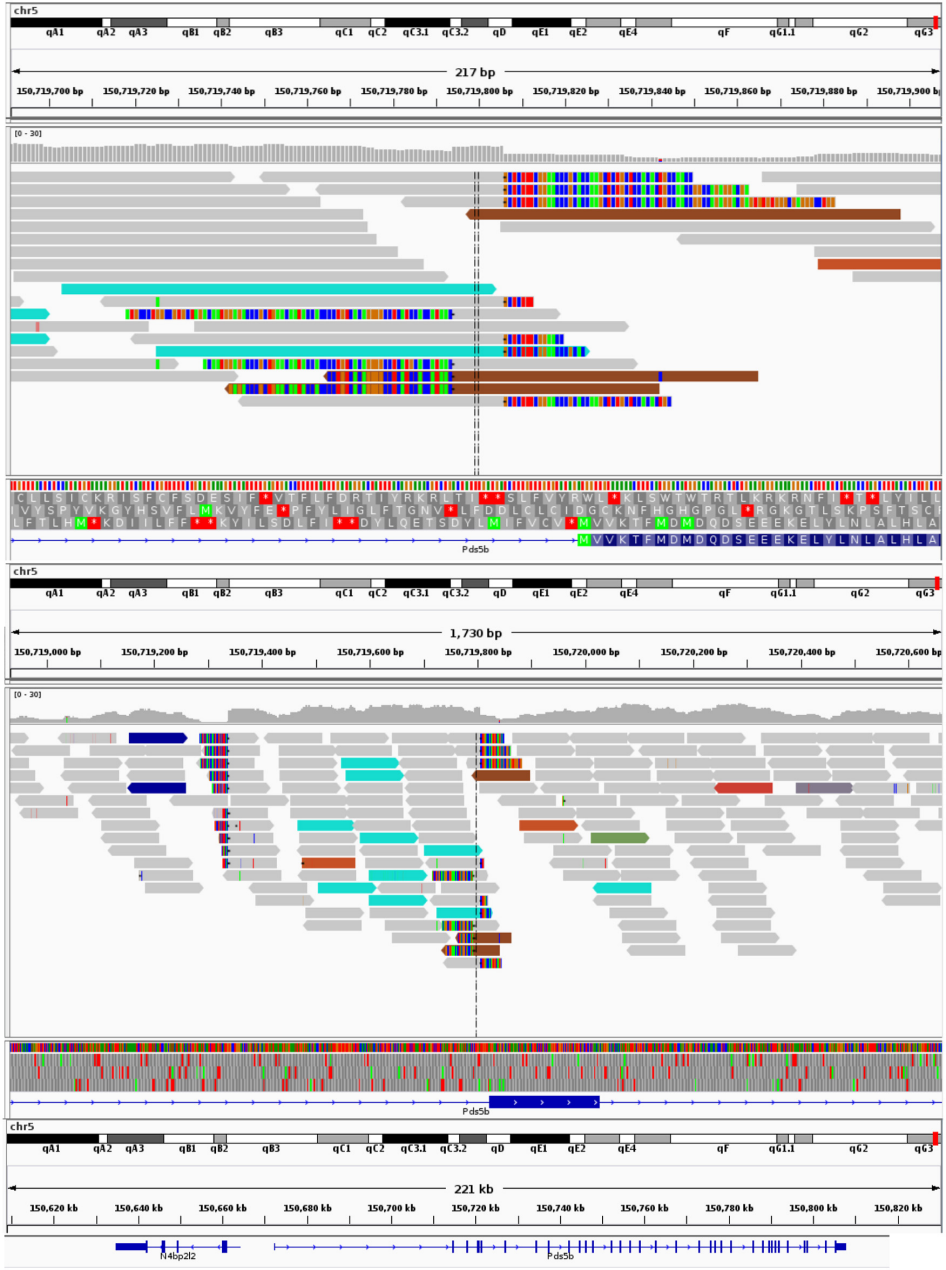
Read alignments surrounding transgene integration. The screenshots from the IGV genome viewer show the integration site of the transgene on chromosome 5. **A**) displays the site and its surroundings. Grey reads are concordantly mapped reads. The colored reads indicate that the other half of the fragment maps discordantly. The teal colored reads are those that have mates on the Her2 transgene. Multi-colored segments at the ends of reads highlight soft-clipped portions of reads. There are three clusters of soft-clipped reads. Two around the integration site, and another block upstream that is unrelated to the integration. **B**) shows the integration in more detail. The proximity to exon 3 of the gene can be seen at the bottom. The right block of soft-clipped reads contains sequence that corresponds to just upstream of the transgene coordinate 4025 (the insert). The left block’s sequence derives from transgene coordinate 2972 and marks the return to chromosome 5 at the end of the concatemer.

**Fig 4:**
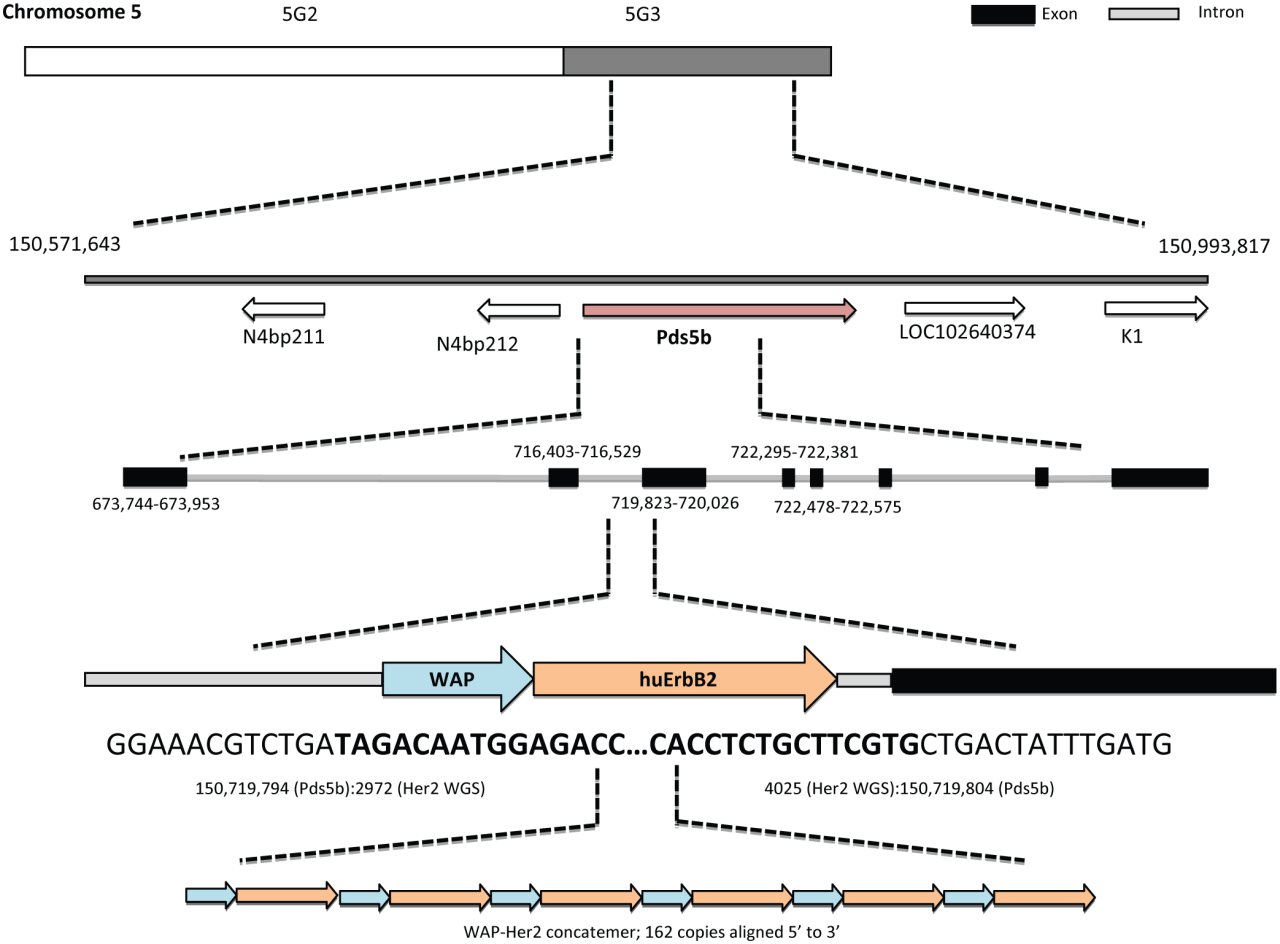
Her2^+/-^ mice harbour 162 copies of the Her 2 transgene in their genome. Genomic DNA from Her2^+/-^ mice was extracted and analysed using whole genome sequencing. The WAP-Her2 transgene had integrated at positions 150719804 (entry) and 150719794 (exit) on chromosome 5, resulting in a 10 nucleotide repeat (from position 150719804–794). A unidirectional concatemer with 162 copies of the WAP-Her2 transgene was found in Her2^+/-^ mice.

The breakpoint detection performed with Socrates predicted 173 fusions in the data. To establish the insertion point we searched for overlap with the transgene sequence within the breakpoint set. There were three fusions that overlapped with the transgene: one from the transgene position 1 to the transgene position 6850, another from chromosome 5 at position 150719804 to the transgene (WAP-Her2) at position 4025, and finally from WAP-Her2 at position 2972 to chromosome 5 at position 150719794. A 10 nt duplication (150719804 to 150719794) was observed at these breakpoints in the host genome. The first fusion was caused by the transgene arranged in a 162 copy concatemer and therefore looping back onto its own start. The latter two breakpoints corresponded to the insertion site of the transgene into the native genome. Using whole genome sequencing, we were able to establish the WAP-Her2 transgene had inserted just 19 nucleotides upstream of exon 3 (150719823) in the Pds5b gene on chromosome 5 (Figs 3 and 4). The whole genome sequencing dataset can be found in the European Nucleotide Archive with accession number PRJEB9805.

**Fig 5:**
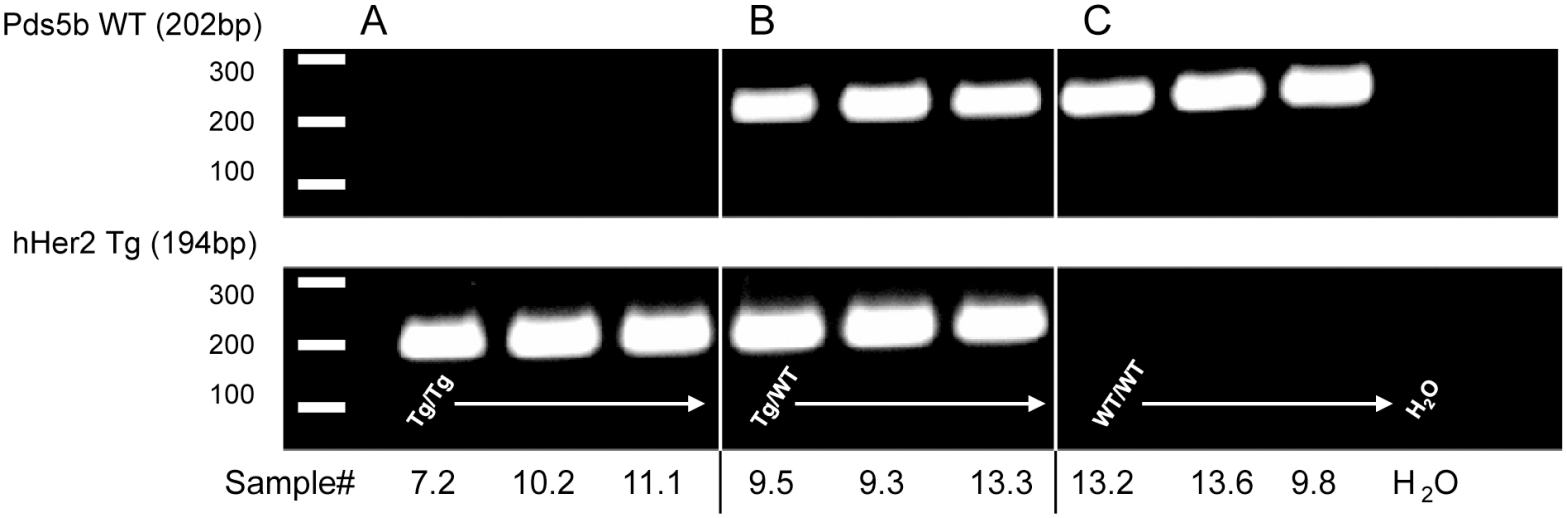
PCR analysis of Her2^+/+^, Her2^+/-^, and WT mice. Genomic DNA from Her2^+/+^, Her2^+/-^ and Her2^-/-^ mice was extracted when mice were sacrificed and PCR amplified to determine the presence of the Her2 transgene and Pds5b gene. The amplified DNA was analysed on a 1% agarose gel. (**A**) The presence of one Her2 band (194bp) signified Her2^+/+^ (Tg/Tg), while (**B**) the presence of both the Her2 band and Pds5b band (202bp) signified Her2^+/-^ (Tg/WT) and (**C**) the presence of one Pds5b band signified Her2^-/-^ (WT/WT) genotypes. Representative gel depicting three of each genotype.

In order to confirm the integration site and distinguish between homozygous and heterozygous mice, we designed primers specific for either the Pds5b gene or the Her2 transgene. Genomic DNA was extracted from embryos at E14 to birth and assessed for the presence of either the Pds5b gene or Her2 transgene by polymerase chain reaction (PCR). A proportion of embryos produced a single Her2 DNA band, indicating their homozygosity (Fig 5A). Her2^+/-^ or Her2^-/-^ mice were phenotypically similar and were accurately genotyped based on the presence of either two bands (Pds5b and Her2) or one band for Pds5b (Fig 5B and 5C).

### WAP-Her2 insertion results in embryonic lethality and a severe developmental phenotype

The insertion of the WAP-Her2 transgene concatemer was found to lie within an intron (19 nucleotides upstream of exon 3) of the Pds5b gene, presumably disrupting and inhibiting normal transcription and translation of Pds5b. Although the RNA splice acceptor site was retained, the insertion of 162 copies of the transgene, constituting approximately 1.1 Mb, likely prevented effective pre-mRNA processing. As heterozygous Her2^+/-^ mice harboured a large number of copies of the Her2 transgene (162 copies), we hypothesised that a further increase in transgene copies in homozygous Her2^+/+^ mice (324 copies) was unlikely to produce an additive effect, and thus have very little effect on the overall phenotype in these mice. Instead, we proposed that two copies of the Her2 concatemer present in Her2^+/+^ mice would completely prohibit normal Pds5b production and function. In addition, we hypothesised that Her2^+/+^ mice would display a phenotype similar to that of *Pds5b* ^-/-^ mice.

Pds5b (also known as APRIN) is a homolog of Pds5, a regulatory factor of the cohesin complex. The cohesin complex is essential in dictating accurate chromosomal segregation during sister chromatid cohesion for cell division, in both mitosis and meiosis [24,32]. Disruption of this process results in dysregulated centromeric cohesion and subsequently an unequal distribution of sister chromatids into daughter cells. The importance of Pds5b in this process has been demonstrated in fetal hepatocytes, where the complete loss of Pds5b resulted in much higher rates of aneuploidy when compared to a loss of Pds5A or wildtype cells alone [26]. As Pds5b^-/-^ models also display a myriad of defects in multiple organs, it is speculated that Pds5b may also play a role in regulating the transcription of a number of genes essential for development and organogenesis [25]. Furthermore, heterozygosity of genomic region containing Pds5b has been detected in a number of tumors and thus Pds5b has been nominated as a potential tumor suppressor gene [33].

While highly conserved, the function and necessity of Pds5b greatly varies between species [34]. *Pds5b* ^-/-^ mice have yielded conflicting results, which may be due to varying levels of expression and subsequent penetrance observed in these models [35]. In one particular study, 75% of *Pds5b* ^-/-^ mice were found to survive to birth but died shortly after. These mice displayed signs of labored breathing, exhibited respiratory distress and had cardiac abnormalities, all of which may have contributed to their early death [25]. The role of Pds5b in organogenesis was apparent, as *Pds5b* ^-/-^ mice presented with signs of growth retardation, including facial dysmorphisms, smaller head and limb structures and abnormal cleft palates. However, other studies using *Pds5b* ^-/-^ have observed a more severe phenotype, with embryonic lethality occurring at the late post-implantation stages (E16.5 to E18.5), with no *Pds5b* ^-/-^ mice surviving until birth (0 out of 500) [26]. In that particular model, loss of both *Pds5b* alleles greatly affected embryonic development and resulted in embryonic death, however no centromeric cohesion defects were observed in *Pds5b* ^+/-^ mouse embryonic fibroblasts (MEFs) [26].

**Fig 6:**
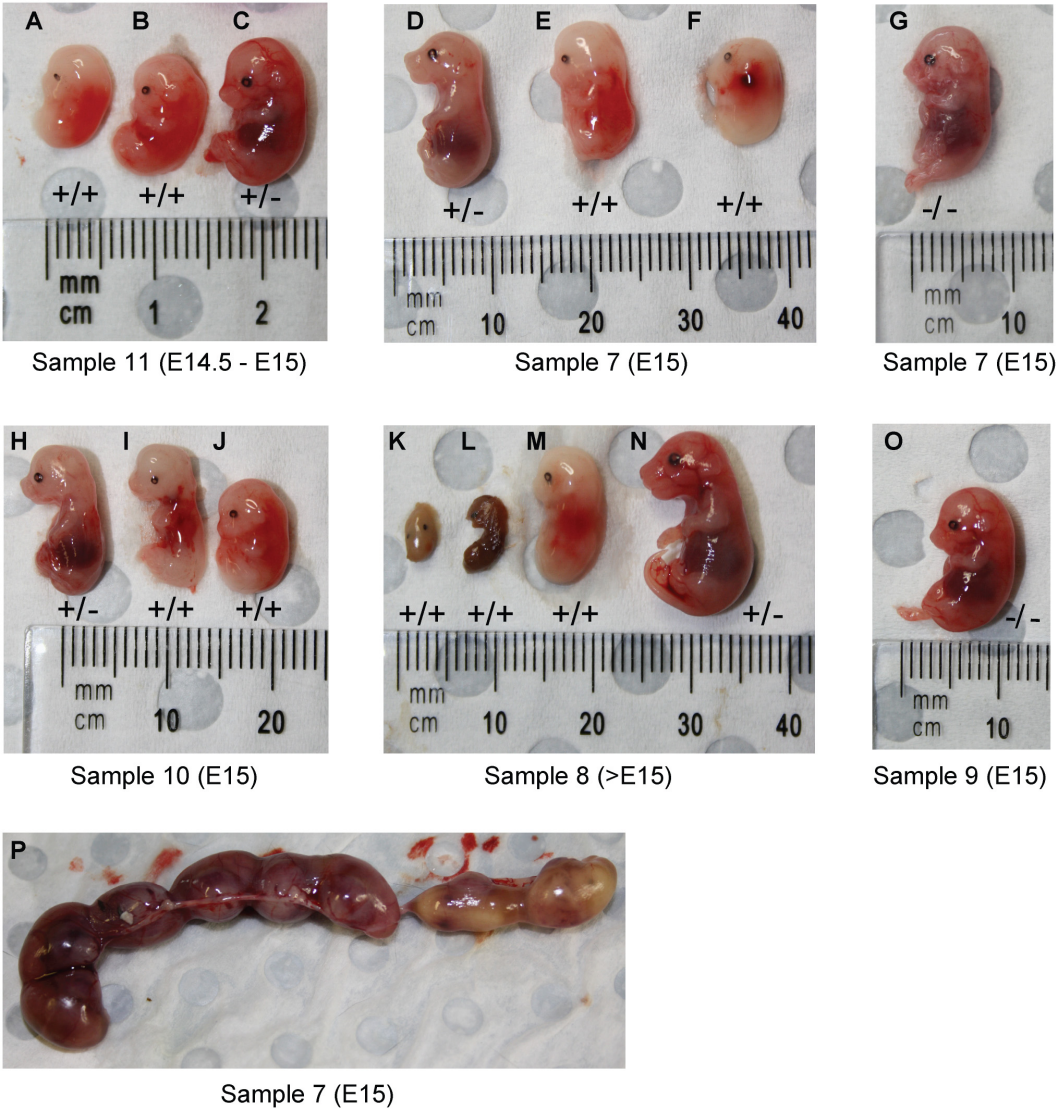
Her2^+/+^ mice display an abnormal phenotype at E15. Her2^+/-^ pregnant females (bred to Her2^+/-^ males) were sacrificed at days E14.5 to >E15 and embryos harvested. **A-O)** Representative images of fetuses. **P)** Amniotic sacs taken from a single female showing distinct yellow appearance of two fetuses in situ. Sample number refers to the litter from which the mice were taken. Mice within the same sample number were taken at the same time point. Genotypes of mice indicate homozygous for the Her2 transgene (+/+), heterozygous for the Her2 transgene (+/-) or wild type (-/-). Genomic DNA was taken and analysed for the expression of both Pds5b and Her2 by PCR to confirm zygosity (see Fig 5).

To determine if the insertion of the Her2 transgene had indeed interrupted normal Pds5b function, we crossed Her2^+/-^ x Her2^+/-^ mice to generate litters of Her2^+/+^, Her2^+/-^ and Her2^-/-^ mice. As developmental defects observed in *Pds5b* ^-/-^ mice were only apparent at E14.5 to E16.5 [25], we harvested embryos at E14 onwards and looked for phenotypic malformations. Surprisingly, we observed a stark difference between Her2^+/+^ mice and Her2^+/-^ or Her2^-/-^ mice. Her2^+/+^ mice taken at E14-E15 displayed obvious developmental defects, were severely underdeveloped and appeared much smaller than either Her2^+/-^ or Her2^-/-^ mice (Fig 6). Her2^+/+^ mice were also jaundiced in colour. In addition, limb and facial formation in these mice was also severely disrupted. No overt difference was observed between Her2^+/-^ and Her2^-/-^ mice (Fig 6D and 6G).

Interestingly, we noted differences between Her2^+/+^ embryos from the same litter in terms of the phenotypic severity (Fig 6K vs 6L vs 6M). While some Her2^+/+^ mice seemed to have developed some facial and limb structure (Fig 6B and 6E), others embryos from the same litter seemed to develop more distinct abnormalities than their littermates (Fig 6A and 6F). As previous studies have also noted variances in the penetrance in the absence of Pds5b [35], we hypothesise a similar effect in the Her2^+/+^ model.

Previous models of *Pds5b* ^-/-^ mice found these null mice were present in a normal Mendelian ratio up until E16.5, with only 75% of *Pds5b* ^-/-^ pups surviving until birth but dying soon after [25]. In contrast, we observed expected Mendelian ratios in embryos up until E15, however no homozygous Her2^+/+^ mice survived to birth (Table 1). Furthermore, we observed extremely mutated embryos at time points past E16 (Fig 6K and 6L), indicating the developmental defects in Her2^+/+^ mice were so severe that it resulted in their resorption.

**Table 1:** Percentages of homozygous and heterozygous Her2 mice at various stages of embryo development.

Age	Genotype			Total
	Her2^-/-^	Her2^+/-^	Her2^+/+^	
E16 to birth	14 (36%)	25 (64%)	0 (0%)	39
E15	7 (17.5%)	22 (55%)	11 (27.5%)	40

Heterozygous Her2^+/-^ mice were interbred to generate litters containing Her2^+/+^, Her2^+/-^ and Her2^-/-^ mice. Embryos were harvested at embryonic day 14–15 (E15) or post E16 to birth. Genomic DNA was extracted and assessed for the presence of the Her2 transgene or intact Pds5b gene. Ratios of Her2^+/+^, Her2^+/-^ and Her2^-/-^ mice were analysed from 11 females with a total of 79 embryos. Her2^+/+^ mice did not survive past E16.

## Discussion

The differential protein expression between oncogenic transformed cells and their cell of origin underpins the basis of antigen-targeted therapies. These therapies profit by selectively targeting proteins uniquely expressed on tumors, leaving the remaining surrounding tissues unaffected. However, such as in the case of the Her2 antigen, tumor cells are also known to up-regulate the expression of proteins found on normal tissues. The generation of the Her2 transgenic mouse model has facilitated the development of anti-Her2 therapies in a competent, and more importantly, clinically relevant model. Previous studies have reported the expression of the human Her2 antigen to be present in both the brain and mammary tissues in this model. Furthermore, DNA vaccination in these mice was sufficient to overcome immune tolerance, and resulted in an increased survival when challenged with Her2 expressing tumors [18].

In the present study, homozygous Her2^+/+^ mice were not viable and resulted in embryonic lethality, however no overt pathologies or abnormalities were observed in their heterozygous littermates. We hypothesised that it was highly unlikely that the increased dosage of the Her2 antigen was responsible for this lethality, but rather that the Her2 transgene had integrated into gene essential for survival. Whole genome sequencing (WGS) was the most efficient and accurate method for determining the transgene integration site in our studies. Prior to WGS, determining the integration point of a transgene required a combination of multiple assays, each laborious and time consuming. Fluorescent in situ hybridisation (FISH) requires the generation of a sequence specific probe, and can only reveal the location of the transgene on a global genome scale. To determine the exact nucleotide location, TAIL-PCR or primer walking would have to be applied [36]. Finally, the number of transgene insertions would have to be assessed using Southern Blot. With WGS the integration is directly observable, given sufficient coverage.

Our results from WGS revealed the WAP-Her2 transgene had formed a long unidirectional concatemer and had integrated at a single position in the genome, upstream of exon 3 of the protein-coding gene, *Pds5b*. The WAP-Her2 concatemer consisted of 162 copies of the transgene, each orientated in a ‘head to tail’ manner. Taking into account the length of the transgene and the high copy number present, we hypothesise the integration of the WAP-Her2 transgene would have isolated the *Pds5b* promoter from the coding sequence downstream of the integration site, and thus effectively disrupted accurate transcription of the *Pds5b* gene. We cannot conclude whether an insertion of this size may have also impacted transcription of the genes flanking the *Pds5b* gene, and thus potentially have contributed to the observed phenotype. However, the similarities between our mice and other models deficient in *Pds5b* lead us to hypothesize the main phenotypic characteristics of this model was due to the dysregulated expression of *Pds5b*.

It was interesting to note both the copy number and orientation of the WAP-Her2 transgene in the Her2 tg mice. Whole genome sequencing analysis revealed 162 copies (in heterozygous mice) were present and moreover, that all 162 copies were ligated together and had in fact integrated into one location. Furthermore, the orientation of each transgene in the transgene polymer was observed to be ligated in a unidirectional manner and always present in a 5’ to 3’ orientation. Other reports characterizing transgenic mice models have observed similar results, where the transgene has been found to concatenate into a long unidirectional concatemer, with each transgene present in a ‘head to tail’ orientation [37–42]. This process occurs prior to insertion into the genome, and is said to be due to the re-circularisation of the linearized transgene DNA. This subsequently results in homologous recombination between the newly formed plasmids, and facilitates the unidirectional orientation observed in these concatemers. While ‘head to head’ and ‘tail to tail’ orientations have been reported, this has only been observed when high concentrations of transgene DNA have been injected [42].

We further substantiated the WGS results by using PCR to detect the presence or absence of an intact genomic *Pds5b*. This confirmed the gene disruption. While further downstream analysis of transcriptional and translational expression of Pds5b, such as RT-PCR or western blot analysis, were not performed, the complete absence of Pds5b at a genomic level in addition to the striking similarities observed between our Her^+/+^ and other published Pds5b^-/-^ models rendered further analysis of downstream expression beyond the scope of the present study, whose aim was to demonstrate the ability of WGS to provide insight into the difficulty in generating homozygous transgenic mice.

The necessity of Pds5b in organogenesis and development has been difficult to accurately assess. Despite the high level of conservation between species, the role of Pds5b and subsequently the phenotype generated from *Pds5* models differs greatly between species. *Pds5* null *S*.*cerevisiae* indicate it plays an integral role in maintaining cohesion and condensation, and is subsequently vital for accurate cell division [43,44]. In contrast, the deletion of *Pds5* in *Schizosaccharomyces pombe* has shown to have little effect on sister chromatid cohesion [45]. Furthermore, the penetrance of the gene deletion within a species can also vary. Deletion of *Pds5b* in mice show varying phenotypes, with some studies observing prenatal or embryonic lethality while others report survival of null mice up until birth but death shortly after. The discrepancies observed may be a result of the methodology and efficiency of the deletion. Indeed, we observed variance in the severity within the same litter in our study. Similar to findings observed by Losada *et al*. [34] the interruption of *Pds5b* in our model resulted in embryonic lethality prior to E16.

In comparison to the severe phenotype observed in Her2^+/+^ mice, we observed no overt developmental abnormalities in Her2^+/-^ mice compared to Her2^-/-^ littermates. This suggests that Pds5b is required in a dose independent manner, and that one functional copy of *Pds5b* is sufficient for normal development. Similar results have been observed in mouse embryonic fibroblasts (MEFs) deficient in one or two alleles for *Pds5b* [26]. Centromeric cohesion remained functional with the loss of one *Pds5b* allele, however, consistent with what we observe in the Her2^+/+^ mice, loss of both *Pds5b* alleles resulted in defective embryonic development leading to prenatal lethality. In addition to the increased frequency of aneuploidy observed in null hepatocytes, Losada *et al*. hypothesise this early lethality may be due to the inability of null cells to maintain mitotic arrest, resulting in an early exit from mitosis and subsequently a reduction in their proliferative capacity [26]. The central aim of our study was to demonstrate the value of whole genome sequencing to determine the likely reason behind the difficulty in generating homozygous transgenic mice. This was clearly demonstrated by a 1.1 MB insertion to disrupt the *Pds5b* gene, and a similar phenotype of lethality and deformities in homozygous embryos to previous studies of *Pds5b*-deficient mice. A full analysis of the *Pds5b*-disrupted mice and comparison to other mouse strains deficient in the *Pds5b* gene was considered beyond the scope of the current manuscript, and therefore in-depth analyses including centromeric cohesion defects and aneuploidy were not performed, but could be the focus of future studies for investigators of *Pds5b* biology.

In considering whether twice the number of copies of Her2 could have contributed to the observed embryonic lethality, we suggest that the requirement of *Pds5b* in a dose-independent manner, in addition to the high copy number of Her2 (162 copies) observed in phenotypically normal heterozygous Her2+/- mice lead us to conclude that the interruption of both alleles of *Pds5b* expression, rather than the increase in Her2 transgene copies (from 162 to 324), resulted in the embryonic lethality of Her2+/+ mice. Other published mouse models have observed the threshold for high transgene expression and subsequent phenotypic deformations to occur at much lower copy numbers (~10–30 copies), therefore we predict the additional 162 copies present in homozygous mice may not have contributed greatly to the embryonic lethality [46,47]. However, further information on the role of twice the number of copies of Her2 could be derived from *Pds5b* transgenic rescue in Her2+/+ mice in future studies.

Interestingly, other models of *Pds5b* ^-/-^ have shown contrasting results to that reported by Losada and what we observe in our study. Milbrandt *et al*. report no observable defects in chromosome cohesion in *Pds5b* ^-/-^ mice, with null mice surviving to birth [25]. These mice display features such as cleft palates, skeletal and bone development malformation, dysmorphic facies and cardiac defects, and ultimately expire soon after birth due to either respiratory or cardiac dysfunction. This may be due to some redundancy between the two isoforms of Pds5 (A and B). While both are integral in telomere cohesion, centromeric cohesion has been shown to be specifically reliant on Pds5b [26]. Using compound homozygotes for both *Pds5a* and *Pds5b*, Milbrandt *et al*. observed early embryonic death, with no double homozygous embryos present at E9.5 [35]. This evidence suggests Pds5a and Pds5b do indeed have redundant functions in organogenesis. Furthermore, compound homozygotes/heterozygotes for either gene resulted in embryonic lethality between days E11.5 to E12.5, with severe growth defects observed in multiple organs, including the enteric nervous system, heart and lens development. However, the absence of one isoform cannot be adequately compensated by the other, as shown in our study. Indeed, other studies using *Pds5b* ^-/-^ mice have shown the expression of Pds5a is independent of Pds5b expression [26], indicating that Pds5a cannot adequately compensate in the absence of Pds5b.

The generation of the human Her2 model has facilitated the development of many Her2 based therapeutics and has proved to be an important tool in immunotherapy. The basal expression of tumor antigens on normal tissue is a major hurdle in immunotherapy, and designing therapeutics that specifically target tumor antigens whilst leaving surrounding tissue unscathed is challenging. The first and only clinical trial involving genetically modified chimeric antigen receptor (CAR) bearing T cells directed towards the Her2 antigen highlighted the importance of on-tumor effects. Low levels of Her2 present on lung epithelial cells were found to trigger the infused CAR T cells, resulting in a cytokine storm and eventuating in the patient’s death [7]. Therefore, future therapeutic designs must take into account possible on-target/off-tumor effects, and strategies to counteract these side effects are essential in order for the progression of anti-Her2 immunotherapies into the clinic. As the Her2 antigen is a self-antigen and not generated *de novo*, in addition to minimising side effects associated with off-tumor targeting, Her2 immunotherapies that engage the hosts’ immune system must also overcome the issue of self-tolerance.

The expression of human Her2 from birth in Her2 mice ensured sufficient immune tolerance to permit Her2^+^ tumor growth, and in addition, allowed for the assessment of autoimmune or on-target/off-tumor effects from therapy. As such, the Her2 model has been utilized extensively in the design and testing of Her2 therapeutics, including those designed to target Her2^+^ breast cancer stem cells [48], Her2 DNA vaccines (in the presence or absence of CD25^hi^ regulatory T cells) [49–51] as well as in adoptive immunotherapy using genetically modified T cells [22,23]. In this study, we further characterise the Her2 model and determine the cause behind the embryonic lethality observed in their homozygosity. Using whole genome sequencing, we determined the transgene integration site to be located within the *Pds5b* gene, a regulator of the cohesin complex and integral for both organogenesis and development. We demonstrate that similar to other *Pds5b* ^-/-^ models, Her2^+/+^ mice are severely underdeveloped, resulting perinatal death and ultimately their resorption from E16 onwards. Our results further contribute to the understanding behind the Her2 transgenic model.

## Supporting Information

S1 Fig Sequence of the WAP-Her2 transgene as found by whole genome sequencing (WGS). The mouse WAP promoter is highlighted in blue, 5’ untranslated region (UTR) of the human ErbB2 cDNA in orange, open reading frame of the human ErbB2 cDNA in pink with the open reading frame (ORF) translated sequence underneath, followed by the 3’ UTR of the human ErbB2 cDNA in yellow. Underlined = truncation of final copy in inserted concatemer and 14 bp of genotyping primer.
(PDF)
