# Supplementary material for: Embryonic Lethality in Homozygous Human Her-2 Transgenic Mice Due to Disruption of the Pds5b Gene
Source: PLoS One. 2015 Sep 3;10(9):e0136817. doi: 10.1371/journal.pone.0136817 (PMC4559457; doi:10.1371/journal.pone.0136817)
Supplement: S1 Fig — The mouse WAP promoter is highlighted in blue, 5’ untranslated region (UTR) of the human ErbB2 cDNA in orange, open reading frame of the human ErbB2 cDNA in pink with the open reading frame (ORF) translated sequence underneath, followed by the 3’ UTR of the human ErbB2 cDNA in yellow. Underlined = truncation of final copy in inserted concatemer and 14 bp of genotyping primer. (PDF) [file pone.0136817.s001.pdf]

Supplementary Figure 1: Sequence of WAP-Her2 transgene

```
                Mouse WAP promoter
   1 CTGGGGATCCTCTAGAGTCGACCTGCAGGCATGCAAGCTTGAATTCTTTCACTGCTAAAACAGGGCGGGAGGAGTCCAGAGCCCTGCCACTGGGTGCAGA 100

 101 ACATGAAGACCCCTTGAGTGGAAAGGGGTTATACAGCTGGACAGTGGTGGCGCACACCTTTAATCCCAGCACTCGGGAGGCAGAGGCAGACGGATCTCTG 200

 201 AGTTCGAGGCCAGCCTGGTCTACAGAATGAGTTCCGGGACAGCCAGGGCCACACAGAGAAACTCTTGTCTCGAAAAACCAAAAAAAAACAAAAAAGGAAA 300

 301 GGGGTTACACAACAGAGACTCAGGTCACAGCTACCCATCACACACAGGATACACATACAAAGGTGTTCACAGGCAGATGAGGAACGAGGAGAAGGGGCTC 400

 401 AAGCAAGGGCCTAAAGTTTCTTTTTTTTTTTTTCTTCTTTTTTTTTTTCCCTGTGGCCTAGAGTTTCAAGAGGCTGAGGACCTAGGCATGAACCAAGAGG 500

 501 GGCCAAACCACTTCAAGAAGCAGGGGGTAGCAGCAGAATCTCACTATCAGCCTTGAGCACAGCTGGGAAGGAGATCCATGGAAACAACCAAGAAAGAGCT 600

 601 GAAAGGGGCTGGAGAGATGGCTCAGCAGTTAAGAGCACTGAGTGCTCTTCCGAAGTCCTAGATTCAAATCCCAGCAACCATATGGTGGGTCACAACCATC 700

 701 TGTAATGAGATCTGATGCCCTCTTCTGGTGTGTCTGAAGACAGCTACAGTGTACTTATGTATAATAAATAAATAAATCTTTAGAAAGGGAGGGGGGGAGA 800

 801 GAGAGAGAGAGAGAGAGAGAGAGAGAGAGAGAGAGAGAGAGAGAGAGAGAGAGAGCTGGAAGAGGGAGATCTGGGAAGTCTGCTGGCTTTATATGCTGACCA 900

 901 TATATAGTCACCTGTGTTTACACACTGTGCTCATCACTTTGAAATCTCAGTGGTTTCTTCTTTGAGCCTGTGTCTGTAAGTTCACCAGGAGAGTGGTACA 1000

1001 TAGGCAAGAATAACAGCCAGTGGGCATAGGACACAGAGTGCATGGGCCCCAGCAAGACTGTAGAGAGAACAGAGCTCTGGCTCCTAAGACATAGGGCCTT 1100

1101 CTGGGAAACTCAAGCAGCCAAGCAACCCTAGCCAGCCCTTTCCTGGTGGCCCTCCTTCTGTTCCAGCAAAGGCGGAAATGGGAACAGGGGTGGAAGCAGA 1200

1201 GCATTGGCAGAGCATAGGTATGACTTAGTCTTGACTAACACAAGCATGGCAGTAGCCTGACAGTGGCCTAAATGTGGGGATGACTGCCTTAGATGGGGAT 1300

1301 GACTGCCTTAGATGGGCATGACTGCCTTAGATGGGGATGACTGCCTTAGATGGGGATGACTGCCTTAGATGGAACAACAAACATCTATGGGCATGCTGTG 1400

1401 GAACACTGGCCCACACACGGAACTGAAGGCACTGGCAATTTCCATAGGGCAGTTAAACCTAAAAGCATGCTCACACTCAACAGGCTGCCGGAATCTCATG 1500

1501 AGACACCTGGAATAGACGAATGTAGAAACAGAGCAGAGAGTTGGTTGCCAAGGTCTGGGGGCTCAGAGGACAAGCAAGAGGCGCGGCTTTCCTTTGGGGC 1600

1601 TGGCATGAAAGGAAATATCGAGGTTACAGCCTGAGAGGGCTTCCCCTGACACTTCGTATTCAAAGAGGCCATGGGCACCAGTGAAGACAAAGGAGTATGG 1700

1701 CCTGCACCACAGGCTGGCNCTGACAGTCAGTAAGCACACAGTCACTCTGGGTCATCCCATCCCCTTCCTTGCAAGAGAAATCAAGGAAATGTCCCGAGAA 1800

1801 CAATGGGGCACAGTGCCAGCAGGACATCTCTTCCTGCCCATGGCACCCTTTGGCACGGTATGGGCCCTTCTGGGAAGGTGGCCTTCCAAATTGCTCTGCA 1900

1901 CAGGCAGCTCCTTTTCAATGTATGCCCGACACTCTCTACATGGAGCAAGCGCCTCCACACTCTTAGAAGAATTTTAGAAAACTCCAGAAAAGCACCAGGA 2000
```

2001 GAAGTCACCCTCAGATGTAGCCCGGACTCGAGCCTTGCTCAAAACCTCCTGTCTTGTTTTCTATGTGACCTGTACAAATTTGGAGCTCAGAATTGCCTTT 2100

2101 GTCTGTGATGGGTTCCAACCCAACCACTCAAAGTGACACTTGTCACATTTGTCACTGATCCTATTTCTTCTTTTTCTGCTCCTTCATTTTCTCCGCTTTC 2200

2201 ATAATAAACAAGTATTACTTTTTAAGTGGGGGAAAAAATGACCACCCTTACAAAGGACTTTTTAAAAATGGCCTCCATTGTGGCCCTTGTTCCTGGCAGC 2300

2301 CTGGGCCTGCTCTCTCTGTGTGGCCAAGAAGGAAGTGTTGTAGCCCATCTAGAGCTGTGCCAGCCTCTTCCCCCACCCCACCCCCAAAGTCTTCCTCCTG 2400

AGE1 **HINDIII** **HUMAN ERBB2 CDNA 5′ UTR**

2401 TGGGTCCTTTAAATGCATCCCAGACACTCAGACAGCCATCAGTCACTTGCCTGACACCGGTACCCAGCTTGGGGCAGCCGCGCGCCCCTTCCCACGGGGC 2500

2501 CCTTTACTGCGCCGCGCGCCCGGCCCCCACCCCTCGCAGCACCCCGCGCCCCGCGCCCTCCCAGCCGGGTCCAGCCGGAGCCATGGGGCCGGAGCCGCAG 2600

ORF

2601 TGAGCACC**ATG**GAGCTGGCGGCCTTGTGCCGCTGGGGGCTCCTCCTCGCCCTCTTGCCCCCCGGAGCCGCGAGCACCCAAGTGTGCACCGGCACAGACAT 2700
M E L A A L C R W G L L L A L L P P G A A S T Q V C T G T D M

2701 GAAGCTGCGGCTCCCTGCCAGTCCCGAGACCCACCTGGACATGCTCCGCCACCTCTACCAGGGCTGCCAGGTGGTGCAGGGAAACCTGGAACTCACCTAC 2800
K L R L P A S P E T H L D M L R H L Y Q G C Q V V Q G N L E L T Y

2801 CTGCCCACCAATGCCAGCCTGTCCTTCCTGCAGGATATCCAGGAGGTGCAGGGCTACGTGCTCATCGCTCACAACCAAGTGAGGCAGGTCCCACTGCAGA 2900
L P T N A S L S F L Q D I Q E V Q G Y V L I A H N Q V R Q V P L Q R

2901 GGCTGCGGATTGTGCGAGGCACCCAGCTCTTTGAGGACAACTATGCCCTGGCCGTGCTAGACAATGGAGACCCGCTGAACAATACCACCCCTGTCACAGG 3000
L R I V R G T Q L F E D N Y A L A V L D N G D P L N N T T P V T G

3001 GGCCTCCCCAGGAGGCCTGCGGGAGCTGCAGCTTCGAAGCCTCACAGAGATCTTGAAAGGAGGGGTCTTGATCCAGCGGAACCCCCAGCTCTGCTACCAG 3100
A S P G G L R E L Q L R S L T E I L K G G V L I Q R N P Q L C Y Q

3101 GACACGATTTTGTGGAAGGACATCTTCCACAAGAACAACCAGCTGGCTCTCACACTGATAGACACCAACCGCTCTCGGGCCTGCCACCCCTGTTCTCCGA 3200
D T I L W K D I F H K N N Q L A L T L I D T N R S R A C H P C S P M

3201 TGTGTAAGGGCTCCCGCTGCTGGGGAGAGAGTTCTGAGGATTGTCAGAGCCTGACGCGCACTGTCTGTGCCGGTGGCTGTGCCCGCTGCAAGGGGCCACT 3300
C K G S R C W G E S S E D C Q S L T R T V C A G G C A R C K G P L

3301 GCCCACTGACTGCTGCCATGAGCAGTGTGCTGCCGGCTGCACGGGCCCCAAGCACTCTGACTGCCTGGCCTGCCTCCACTTCAACCACAGTGGCATCTGT 3400
P T D C C H E Q C A A G C T G P K H S D C L A C L H F N H S G I C

3401 GAGCTGCACTGCCCAGCCCTGGTCACCTACAACACAGACACGTTTGAGTCCATGCCCAATCCCGAGGGCCGGTATACATTCGGCGCCAGCTGTGTGACTG 3500
E L H C P A L V T Y N T D T F E S M P N P E G R Y T F G A S C V T A

```
3501 CCTGTCCCTACAACTACCTTTCTACGGACGTGGGATCCTGCACCCTCGTCTGCCCCCTGCACAACCAAGAGGTGACAGCAGAGGATGGAACACAGCGGTG 3600
       C  P  Y  N  Y  L  S  T  D  V  G  S  C  T  L  V  C  P  L  H  N  Q  E  V  T  A  E  D  G  T  Q  R  C

3601 TGAGAAGTGCAGCAAGCCCTGTGCCCGAGTGTGCTATGGTCTGGGCATGGAGCACTTGCGAGAGGTGAGGGCAGTTACCAGTGCCAATATCCAGGAGTTT 3700
      E  K  C  S  K  P  C  A  R  V  C  Y  G  L  G  M  E  H  L  R  E  V  R  A  V  T  S  A  N  I  Q  E  F

3701 GCTGGCTGCAAGAAGATCTTTGGGAGCCTGGCATTTCTGCCGGAGAGCTTTGATGGGGACCCAGCCTCCAACACTGCCCCGCTCCAGCCAGAGCAGCTCC 3800
     A  G  C  K  K  I  F  G  S  L  A  F  L  P  E  S  F  D  G  D  P  A  S  N  T  A  P  L  Q  P  E  Q  L  Q

3801 AAGTGTTTGAGACTCTGGAAGAGATCACAGGTTACCTATACATCTCAGCATGGCCGGACAGCCTGCCTGACCTCAGCGTCTTCCAGAACCTGCAAGTAAT 3900
       V  F  E  T  L  E  E  I  T  G  Y  L  Y  I  S  A  W  P  D  S  L  P  D  L  S  V  F  Q  N  L  Q  V  I

3901 CCGGGGACGAATTCTGCACAATGGCGCCTACTCGCTGACCCTGCAAGGGCTGGGCATCAGCTGGCTGGGGCTGCGCTCACTGAGGGAACTGGGCAGTGGA 4000
      R  G  R  I  L  H  N  G  A  Y  S  L  T  L  Q  G  L  G  I  S  W  L  G  L  R  S  L  R  E  L  G  S  G

4001 CTGGCCCTCATCCACCATAACACCCACCTCTGCTTCGTGCACACGGTGCCCTGGGACCAGCTCTTTCGGAACCCGCACCAAGCTCTGCTCCACACTGCCA 4100
     L  A  L  I  H  H  N  T  H  L  C  F  V  H  T  V  P  W  D  Q  L  F  R  N  P  H  Q  A  L  L  H  T  A  N

4101 ACCGGCCAGAGGACGAGTGTGTGGGCGAGGGCCTGGCCTGCCACCAGCTGTGCGCCCGAGGGCACTGCTGGGGTCCAGGGCCCACCCAGTGTGTCAACTG 4200
       R  P  E  D  E  C  V  G  E  G  L  A  C  H  Q  L  C  A  R  G  H  C  W  G  P  G  P  T  Q  C  V  N  C

4201 CAGCCAGTTCCTTCGGGGCCAGGAGTGCGTGGAGGAATGCCGAGTACTGCAGGGGCTCCCCAGGGAGTATGTGAATGCCAGGCACTGTTTGCCGTGCCAC 4300
      S  Q  F  L  R  G  Q  E  C  V  E  E  C  R  V  L  Q  G  L  P  R  E  Y  V  N  A  R  H  C  L  P  C  H

4301 CCTGAGTGTCAGCCCCAGAATGGCTCAGTGACCTGTTTTGGACCGGAGGCTGACCAGTGTGTGGCCTGTGCCCACTATAAGGACCCTCCCTTCTGCGTGG 4400
     P  E  C  Q  P  Q  N  G  S  V  T  C  F  G  P  E  A  D  Q  C  V  A  C  A  H  Y  K  D  P  P  F  C  V  A

4401 CCCGCTGCCCCAGCGGTGTGAAACCTGACCTCTCCTACATGCCCATCTGGAAGTTTCCAGATGAGGAGGGCGCATGCCAGCCTTGCCCCATCAACTGCAC 4500
       R  C  P  S  G  V  K  P  D  L  S  Y  M  P  I  W  K  F  P  D  E  E  G  A  C  Q  P  C  P  I  N  C  T

4501 CCACTCCTGTGTGGACCTGGATGACAAGGGCTGCCCCGCCGAGCAGAGAGCCAGCCCTCTGACGTCCATCATCTCTGCGGTGGTTGGCATTCTGCTGGTC 4600
      H  S  C  V  D  L  D  D  K  G  C  P  A  E  Q  R  A  S  P  L  T  S  I  I  S  A  V  V  G  I  L  L  V

4601 GTGGTCTTGGGGGTGGTCTTTGGGATCCTCATCAAGCGACGGCAGCAGAAGATCCGGAAGTACACGATGCGGAGACTGCTGCAGGAAACGGAGCTGGTGG 4700
     V  V  L  G  V  V  F  G  I  L  I  K  R  R  Q  Q  K  I  R  K  Y  T  M  R  R  L  L  Q  E  T  E  L  V  E

4701 AGCCGCTGACACCTAGCGGAGCGATGCCCAACCAGGCGCAGATGCGGATCCTGAAAGAGACGGAGCTGAGGAAGGTGAAGGTGCTTGGATCTGGCGCTTT 4800
       P  L  T  P  S  G  A  M  P  N  Q  A  Q  M  R  I  L  K  E  T  E  L  R  K  V  K  V  L  G  S  G  A  F

4801 TGGCACAGTCTACAAGGGCATCTGGATCCCTGATGGGGAGAATGTGAAAATTCCAGTGGCCATCAAAGTGTTGAGGGAAAACACATCCCCCAAAGCCAAC 4900
      G  T  V  Y  K  G  I  W  I  P  D  G  E  N  V  K  I  P  V  A  I  K  V  L  R  E  N  T  S  P  K  A  N
```

```
4901 AAAGAAATCTTAGACGAAGCATACGTGATGGCTGGTGTGGGCTCCCCATATGTCTCCCGCCTTCTGGGCATCTGCCTGACATCCACGGTGCAGCTGGTGA 5000
     K  E  I  L  D  E  A  Y  V  M  A  G  V  G  S  P  Y  V  S  R  L  L  G  I  C  L  T  S  T  V  Q  L  V  T

5001 CACAGCTTATGCCCTATGGCTGCCTCTTAGACCATGTCCGGGAAAACCGCGGACGCCTGGGCTCCCAGGACCTGCTGAACTGGTGTATGCAGATTGCCAA 5100
       Q  L  M  P  Y  G  C  L  L  D  H  V  R  E  N  R  G  R  L  G  S  Q  D  L  L  N  W  C  M  Q  I  A  K

5101 GGGGATGAGCTACCTGGAGGATGTGCGGCTCGTACACAGGGACTTGGCCGCTCGGAACGTGCTGGTCAAGAGTCCCAACCATGTCAAAATTACAGACTTC 5200
      G  M  S  Y  L  E  D  V  R  L  V  H  R  D  L  A  A  R  N  V  L  V  K  S  P  N  H  V  K  I  T  D  F

5201 GGGCTGGCTCGGCTGCTGGACATTGACGAGACAGAGTACCATGCAGATGGGGGCAAGGTGCCCATCAAGTGGATGGCGCTGGAGTCCATTCTCCGCCGGC 5300
     G  L  A  R  L  L  D  I  D  E  T  E  Y  H  A  D  G  G  K  V  P  I  K  W  M  A  L  E  S  I  L  R  R  R

5301 GGTTCACCCACCAGAGTGATGTGTGGAGTTATGGTGTGACTGTGTGGGAGCTGATGACTTTTGGGGCCAAACCTTACGATGGGATCCCAGCCCGGGAGAT 5400
       F  T  H  Q  S  D  V  W  S  Y  G  V  T  V  W  E  L  M  T  F  G  A  K  P  Y  D  G  I  P  A  R  E  I

5401 CCCTGACCTGCTGGAAAAGGGGGAGCGGCTGCCCCAGCCCCCCATCTGCACCATTGATGTCTACATGATCATGGTCAAATGTTGGATGATTGACTCTGAA 5500
      P  D  L  L  E  K  G  E  R  L  P  Q  P  P  I  C  T  I  D  V  Y  M  I  M  V  K  C  W  M  I  D  S  E

5501 TGTCGGCCAAGATTCCGGGAGTTGGTGTCTGAATTCTCCCGCATGGCCAGGGACCCCCAGCGCTTTGTGGTCATCCAGAATGAGGACTTGGGCCCAGCCA 5600
     C  R  P  R  F  R  E  L  V  S  E  F  S  R  M  A  R  D  P  Q  R  F  V  V  I  Q  N  E  D  L  G  P  A  S

5601 GTCCCTTGGACAGCACCTTCTACCGCTCACTGCTGGAGGACGATGACATGGGGGACCTGGTGGATGCTGAGGAGTATCTGGTACCCCAGCAGGGCTTCTT 5700
       P  L  D  S  T  F  Y  R  S  L  L  E  D  D  D  M  G  D  L  V  D  A  E  E  Y  L  V  P  Q  Q  G  F  F

5701 CTGTCCAGACCCTGCCCCGGGCGCTGGGGGCATGGTCCACCACAGGCACCGCAGCTCATCTACCAGGAGTGGCGGTGGGGACCTGACACTAGGGCTGGAG 5800
      C  P  D  P  A  P  G  A  G  G  M  V  H  H  R  H  R  S  S  S  T  R  S  G  G  G  D  L  T  L  G  L  E

5801 CCCTCTGAAGAGGAGGCCCCCAGGTCTCCACTGGCACCCTCCGAAGGGGCTGGCTCCGATGTATTTGATGGTGACCTGGGAATGGGGGCAGCCAAGGGGC 5900
     P  S  E  E  E  A  P  R  S  P  L  A  P  S  E  G  A  G  S  D  V  F  D  G  D  L  G  M  G  A  A  K  G  L

5901 TGCAAAGCCTCCCCACACATGACCCCAGCCCTCTACAGCGGTACAGTGAGGACCCCACAGTACCCCTGCCCTCTGAGACTGATGGCTACGTTGCCCCCCT 6000
       Q  S  L  P  T  H  D  P  S  P  L  Q  R  Y  S  E  D  P  T  V  P  L  P  S  E  T  D  G  Y  V  A  P  L

6001 GACCTGCAGCCCCCAGCCTGAATATGTGAACCAGCCAGATGTTCGGCCCCAGCCCCCTTCGCCCCGAGAGGGCCCTCTGCCTGCTGCCCGACCTGCTGGT 6100
      T  C  S  P  Q  P  E  Y  V  N  Q  P  D  V  R  P  Q  P  P  S  P  R  E  G  P  L  P  A  A  R  P  A  G

6101 GCCACTCTGGAAAGGCCCAAGACTCTCTCCCCAGGGAAGAATGGGGTCGTCAAAGACGTTTTTGCCTTTGGGGGTGCCGTGGAGAACCCCGAGTACTTGA 6200
     A  T  L  E  R  P  K  T  L  S  P  G  K  N  G  V  V  K  D  V  F  A  F  G  G  A  V  E  N  P  E  Y  L  T

6201 CACCCCAGGGAGGAGCTGCCCCTCAGCCCCACCCTCCTCCTGCCTTCAGCCCAGCCTTCGACAACCTCTATTACTGGGACCAGGACCCACCAGAGCGGGG 6300
       P  Q  G  G  A  A  P  Q  P  H  P  P  P  A  F  S  P  A  F  D  N  L  Y  Y  W  D  Q  D  P  P  E  R  G
```

```
                                                                                                       3′ UTR
6301 GGCTCCACCCAGCACCTTCAAAGGGACACCTACGGCAGAGAACCCAGAGTACCTGGGTCTGGACGTGCCAGTGTGAACCAGAAGGCCAAGTCCGCAGAAG 6400
       A   P   P   S   T   F   K   G   T   P   T   A   E   N   P   E   Y   L   G   L   D   V   P   V   STOP

6401 CCCTGATGTGTCCTCAGGGAGCAGGGAAGGCCTGACTTCTGCTGGCATCAAGAGGTGGGAGGGCCCTCCGACCACTTCCAGGGGAACCTGCCATGCCAGG 6500

6501 AACCTGTCCTAAGGAACCTTCCTTCCTGCTTGAGTTCCCAGATGGCTGGAAGGGGTCCAGCCTCGTTGGAAGAGGAACAGCACTGGGGAGTCTTTGTGGA 6600

6601 TTCTGAGGCCCTGCCCAATGAGACTCTAGGGTCCAGTGGATGCCACAGCCCAGCTTGGCCCTTTCCTTCCAGATCCTGGGTACTGAAAGCCTTAGGGAAG 6700

6701 CTGGCCTGAGAGGGGAAGCGGCCCTAAGGGAGTGTCTAAGAACAAAAGCGACCCATTCAGAGACTGTCCCTGAAACCTAGTACTGCCCCCCATGAGGAAG 6800

            BSRD1
6801 GAACAGCAATGGTGTCAGTATCCAGGCTTTGTACAGAGTGCTTTTCTGTTTAGTTTTTACTTTTTTTGTTTTGTTTTTTTAAAG 6884
```
